# Towards A Microbead Occlusion Model of Glaucoma for a Non-Human Primate

**DOI:** 10.1038/s41598-019-48054-y

**Published:** 2019-08-09

**Authors:** Wendi S. Lambert, Brian J. Carlson, Purnima Ghose, Victoria D. Vest, Vincent Yao, David J. Calkins

**Affiliations:** The Vanderbilt Eye Institute, Vanderbilt University Medical Center, Nashville, TN 37232-0654 USA

**Keywords:** Retina, Neurodegeneration, Animal disease models, Retina, Neurodegeneration

## Abstract

Glaucoma is a group of optic neuropathies associated with aging and sensitivity to intraocular pressure (IOP). The disease causes vision loss through the degeneration of retinal ganglion cell neurons and their axons in the optic nerve. Using an inducible model of glaucoma, we elevated IOP in the squirrel monkey (*Saimiri boliviensis*) using intracameral injection of 35 μm polystyrene microbeads and measured common pathogenic outcomes in the optic projection. A 42% elevation in IOP over 28 weeks reduced anterograde transport of fluorescently-labeled cholera toxin beta from retina to the lateral geniculate nucleus (60% decrease), and to the superior colliculus (49% decrease). Pressure also reduced survival of ganglion cellaxons in the optic nerve by 22%. The same elevation caused upregulation of proteins associated with glaucomatous neurodegeneration in the retina and optic nerve, including complement 1q, interleukin 6, and brain-derived neurotrophic factor. That axon degeneration in the nerve lagged deficits in anterograde transport is consistent with progression in rodent models, while the observed protein changes also occur in tissue from human glaucoma patients. Thus, microbead occlusion in a non-human primate with a visual system similar to our own represents an attractive model to investigate neurodegenerative mechanisms and therapeutic interventions for glaucoma.

## Introduction

Glaucoma, a group of optic neuropathies associated with aging and sensitivity to intraocular pressure (IOP), is the leading cause of irreversible blindness worldwide^1^. Glaucoma involves early dysfunction with subsequent degeneration of retinal ganglion cells and their axons due to IOP-related stress conveyed at the optic nerve head^2,3^. Since the early 1980’s, animal models have been the gold standard for studying progression and pathology in glaucoma. Some models are inducible, using artificial means to elevate ocular pressure (e.g. microbead injection, hypertonic saline injection, laser photocoagulation); others are naturally-occurring, such as the DBA2J mouse^4–8^. Combining observations from models of glaucoma and human glaucomatous samples has led to a better understanding of the progression of this disease, the potential mechanisms involved, and of pathways key to ganglion cell survival or loss^9–14^. Additionally, different glaucoma models have allowed testing and refining of therapeutic targets and drug delivery systems^15–19^.

The squirrel monkey (SM, *Saimiri*) is a new world primate well-suited for animal studies that attempt to model the human visual system in health and disease. Due to their size, temperament and adaptability, SMs represent a convenient model, with eye, optic projection, and brain similar in structure and function to that of other non-human primates, including old-world primate species like macaque or the great apes^20–23^. In terms of translational medicine, the SM eye has served as a model system for studies of dry eye, choroidal neovascularization, color blindness, and aqueous humor flow – which is seminal in the pathogenesis of glaucoma^23–27^. Similarly, the retina and optic nerve of the SM are extremely similar in structure and function to the human, and so are useful in examining pathology associated with eye disease^28–30^.

Several years ago, we and others developed a model of glaucoma in which IOP elevation is induced through injection of polystyrene microbeads into the anterior segment of the eye^4,5,31^. This elevation in pressure allows for testing in the short term how glaucomatous-related stress affects the health of the retina, optic nerve and brain^32–34^. Here, we expand this model to SMs because the structure of the anterior segment and the physiology of aqueous flow are highly similar to the human^28–30^, and thus are an appropriate next step in studies of mechanisms of progression in glaucoma. Additionally, examining neurodegeneration in SMs using this inducible model will help validate therapeutic targets identified in rodents as most promising for patients suffering with glaucoma. In this study, we elevated IOP in SMs using microbead occlusion for up to 28 weeks, which resulted in anterograde transport deficits in the lateral geniculate nucleus and superior colliculus, axon loss in the optic nerve, and the expression of proteins implicated in glaucoma pathogenesis. Microbead occlusion in SMs induces degenerative changes similar to those observed in rodent models and in human glaucoma patients, making it an accessible model to investigate degenerative mechanisms and therapeutic interventions.

## Results

### Elevation of IOP following microbead injection

**Figure 1:**
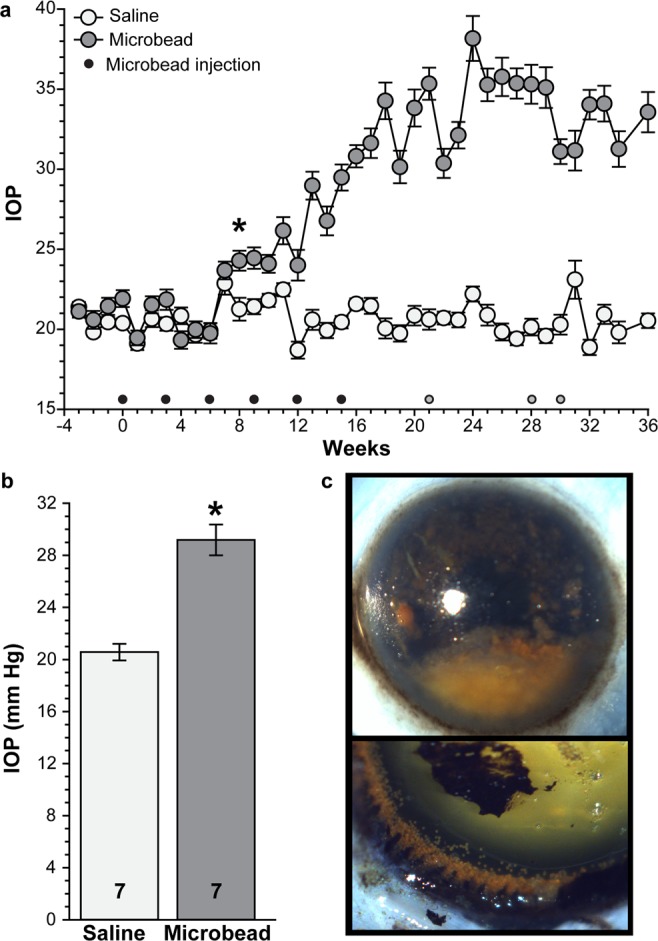
Microbead injection increases SM IOP. (**a**) IOP measurements in awake SMs prior to and after intracameral microbead injection (dots). Black dots represent MOM injections required to elevate IOP. Grey dots represent re-injection (one SM/dot) when IOP in that SM appeared to be decreasing. *p < 0.008 from week 8 to end of study, n = 7 eyes for saline and microbead. (**b**) Bar graph showing mean IOP in saline- and microbead-injected eyes. *p = 0.00003, n indicated in bars. (**c**) Representative post-mortem images of microbeads in the anterior chamber (top) and along the iridocorneal angle following dissection of the cornea (bottom). Data are expressed as mean ± SEM. Statistical comparisons were made using two-sided t-tests.

Baseline IOP over an initial four-week period ranged from 19.8 to 21.4 mm Hg with an average of 20.9 ± 0.7 mm Hg (n = 7 for saline and microbead); this range is slightly higher than previously reported values obtained under anesthesia^35^. Injection of saline into the anterior chamber of control eyes had little to no effect on IOP, while IOP in microbead-injected eyes (gray circles) began to increase after three injections (Fig. 1a, black dots). Pressure ranged from 18.7 to 23.1 mm Hg in saline-injected eyes and from 19.3 to 38.2 mm Hg in microbead-injected eyes. Elevation of IOP occurred eight weeks after the initial microbead injection relative to saline-injected eyes and remained elevated for an additional 28 weeks (10.5–82.2% elevation, 51.8% mean elevation, p < 0.008, n = 7 for saline and microbead). An experimental eye received one additional microbead injection if IOP decreased over three consecutive weeks; three SMs received an additional microbead injection at the times indicated. Over the entire experimental period (36 weeks total), microbead injection induced an IOP elevation of 41.8%, corresponding to a mean IOP of 29.2 ± 1.2 mm Hg compared to 20.6 ± 0.6 mm Hg in control eyes (Fig. 1b; p = 0.00003, n = 7 for saline and microbead). *In vivo*, microbeads appeared to cluster within the anterior chamber, but upon examination post-mortem were observed deposited along the iridocorneal angle (Fig. 1c). We analyzed subsequent outcome measures (anterograde transport, optic nerve degeneration, protein expression) at 36 weeks post-initial microbead injection.

### Microbead-induced deficits in anterograde transport

**Figure 2:**
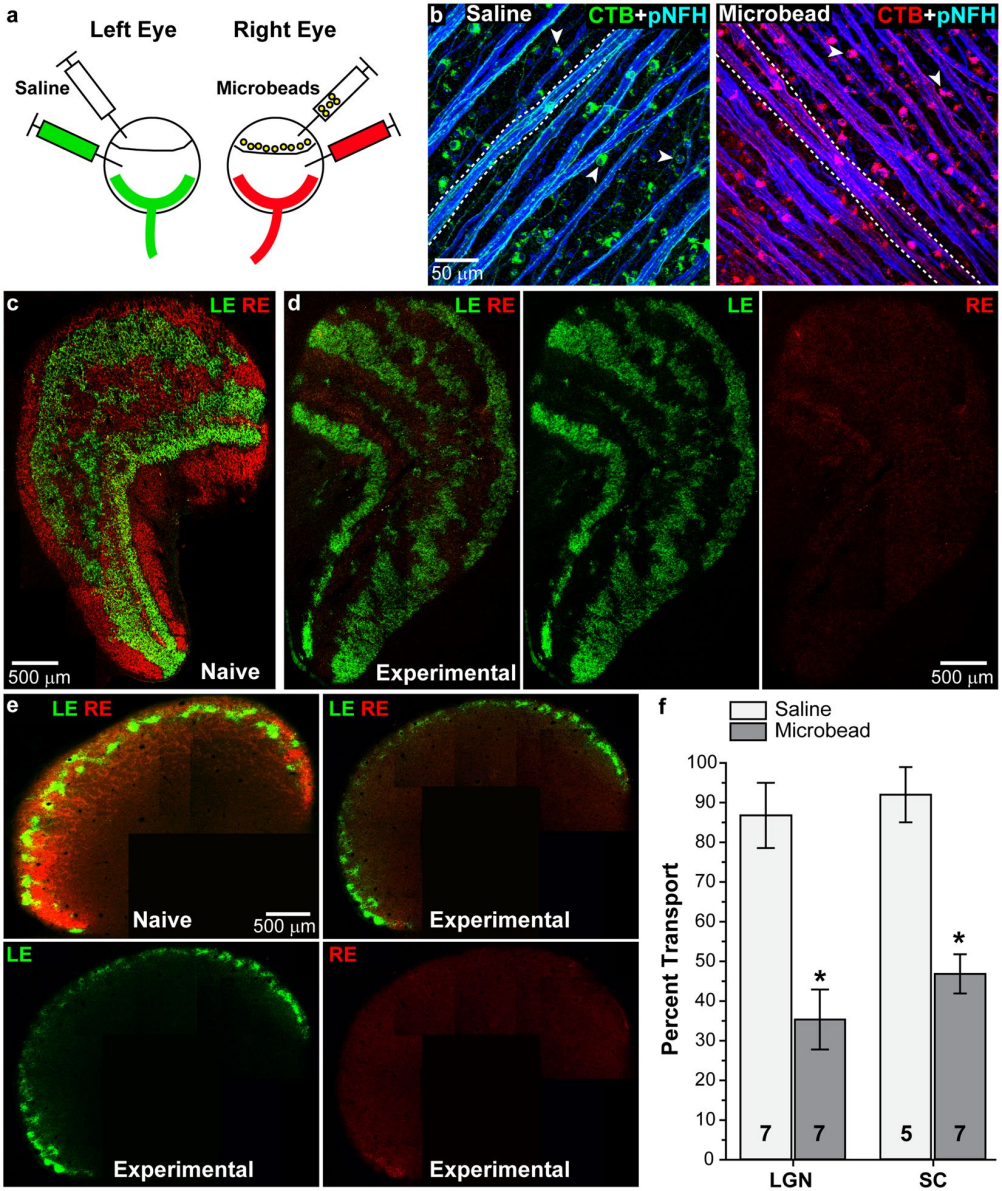
Elevated IOP following microbead injection decreases anterograde transport in SMs. (**a**) Schematic showing microbead and CTB injection paradigm. Left eyes received saline and CTB-488 (green) while right eyes received microbeads and CTB-594 (red). (**b**) Whole-mounted retinas from saline- and microbead-injected retinas showing CTB (green or red) uptake by RGC somas (arrowheads) and transport in axons (dotted lines). Phosphorylated neurofilament heavy (pNFH, blue) expression is shown in RGC somas and axons (**c**,**d**) Coronal section of a LGN from a naïve SM or an experimental SM. CTB transported from the left eye (LE, green) and the right eye (RE, red) is shown. (**e**) Coronal sections of a SC from a naïve or an experimental SM. (**f**) Bar graph showing percent intact transport to the LGN and SC from saline- and microbead-injected eyes. *p < 0.0006; n indicated in bars. Data are expressed as mean ± SEM. Statistical comparisons were made using two-sided t-tests.

Deficits in anterograde transport to ganglion cell axonal targets in subcortical structures is an early response to elevated IOP in rodent models of glaucoma that precedes frank axon degeneration in the optic nerve; both precede ganglion cell body loss in the retina^2,13,36^. While in rodents the majority (~95%) of ganglion cells axons project contralaterally, ganglion cells in SMs and other primates project ipsilaterally and contralaterally in about equal (50:50) proportion^37^. To test how elevated IOP influences anterograde transport in the SM, we injected fluorescently-labeled cholera toxin B (CTB) into the vitreous chamber of both saline and microbead eyes one week prior to sacrifice (Fig. 2a). Since CTB transport along axons depends on active uptake by ganglion cells, we assessed initial uptake by imaging whole-mounted retinas (Fig. 2b). Colocalization of CTB (green in left eye, red in right eye) and phosphorylated neurofilament (heavy-chain, blue) was similarly strong in ganglion cell bodies and axons from both saline- and microbead-injected eyes. Similar numbers of labeled RGCs were observed in both saline-and microbead-injected eyes suggesting no or minimal RGC loss at the time of CTB injection (Fig. 2b). Images from representative sections through the lateral geniculate nucleus (LGN, the primary ganglion cell subcortical target in primates) show depleted anterograde transport from an eye with elevated IOP (Fig. 2d, red CTB) compared to transport from a saline (Fig. 2d, green CTB) or naïve eye (Fig. 2c). Elevation of IOP induced similar transport deficits in sections of superior colliculus (SC, the most distal projection site receiving input from far fewer ganglion cells) compared to transport from control or naïve eyes (Fig. 2e). When quantified across serial sections, anterograde transport from saline-injected eyes was nearly complete in both LGN and SC (86.8 ± 8.2% intact, and 92.0 ± 6.9% intact, respectively), while elevated IOP in microbead-injected eyes reduced transport to the LGN by 59.2%, resulting in 35.4 ± 7.6% intact transport (p = 0.0006, n = 7 for saline and microbead). Likewise, transport to the SC decreased 49.1%, leaving 46.8 ± 4.9% intact transport (p = 0.0003, n = 5 for saline and 7 for microbead). We observed no difference in total cross-section area between saline-injected and microbead-injected LGN or SC (p > 0.2), indicating that transport deficits precede frank degeneration of RGC relay targets, similar to our findings in other models of glaucoma^12^.

### Optic nerve axon degeneration following microbead injection

**Figure 3:**
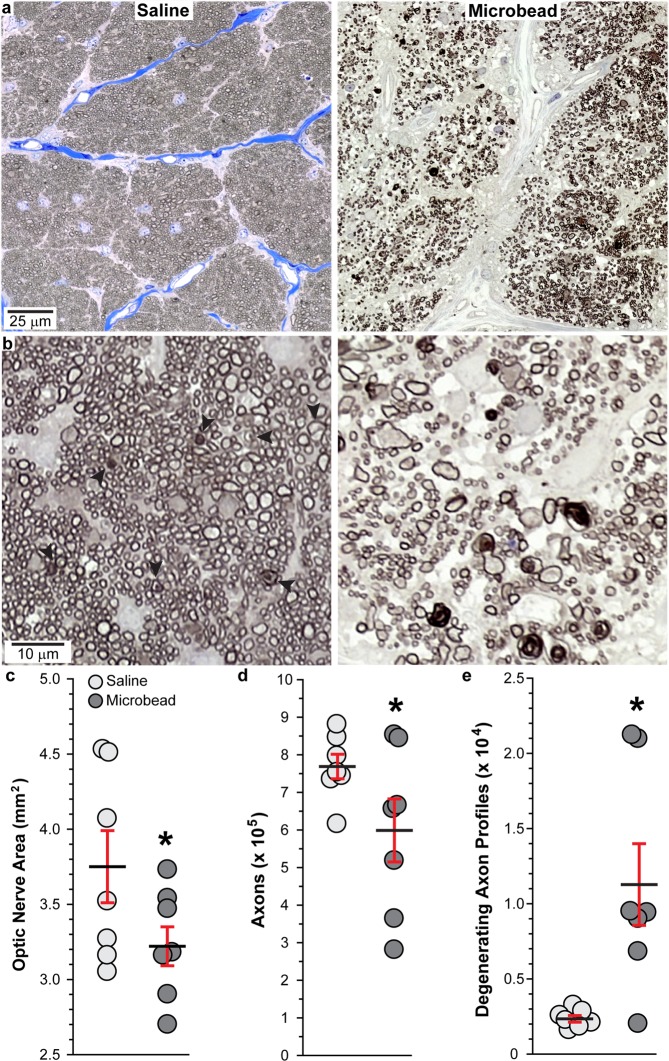
Elevated IOP decreases RGC axons in SM optic nerve. (**a**) Representative images of SM optic nerves following saline or microbead injection. (**b**) Optic nerve images from microbead-injected eyes showing degenerating axon profiles surrounded by intact axons (arrowheads, left) and severe degeneration where few intact axons remain (right). (**c**–**e**) Scatter plots showing mean area of optic nerves, mean RGC axon number, and mean degenerating axon profiles from saline- and microbead-injected SM eyes. Black lines indicate mean, red bars indicate SEM. *p = 0.038 (**c**), p = 0.042 (**d**), and p = 0.007 (**e**); n = 7 for each group. Data are expressed as mean ± SEM. Statistical comparisons were made using two-sided t-tests.

Next, we evaluated how elevated IOP influenced ganglion cell axon survival in the optic nerve. Representative images show that optic nerves from microbead-injected eyes displayed areas with disorganized axon fascicles, increased numbers of degenerating axon profiles, and rampant hypertrophy of astrocyte processes (Fig. 3a), indicative of axon degeneration and astrocyte hypertrophy^38,39^. Higher magnification images of degenerating axon profiles (arrowheads) in optic nerves from microbead-injected eyes are shown in Fig. 3b. When quantified, optic nerves from saline-injected eyes varied in cross-sectional area between 3.09 to 4.54 mm^2^, with a mean area of 3.75 ± 0.24 mm^2^ (Fig. 3c). Microbead-induced IOP elevation reduced optic nerve area by 14.1%, with a range of 2.71–3.66 mm^2^ and mean of 3.22 ± 0.13 mm^2^ (p = 0.038, n = 7 for saline and microbead). Elevated IOP reduced axon number 22.1% from 769,059.4 ± 32,646.7 axons to 598,964.7 ± 83,870.3 axons (Fig. 3d, p = 0.042, n = 7 for saline and microbead). Axon number in saline-injected eyes was similar to previously published axons counts for SMs (821,529 to 879,529 axons)^40^. Finally, the number of degenerating axon profiles (Fig. 3e) increased more than 300% with elevated IOP when compared to saline nerves (p = 0.007, n = 7 for saline and microbead).

### Expression of proteins implicated in glaucoma following microbead injection

**Figure 4:**
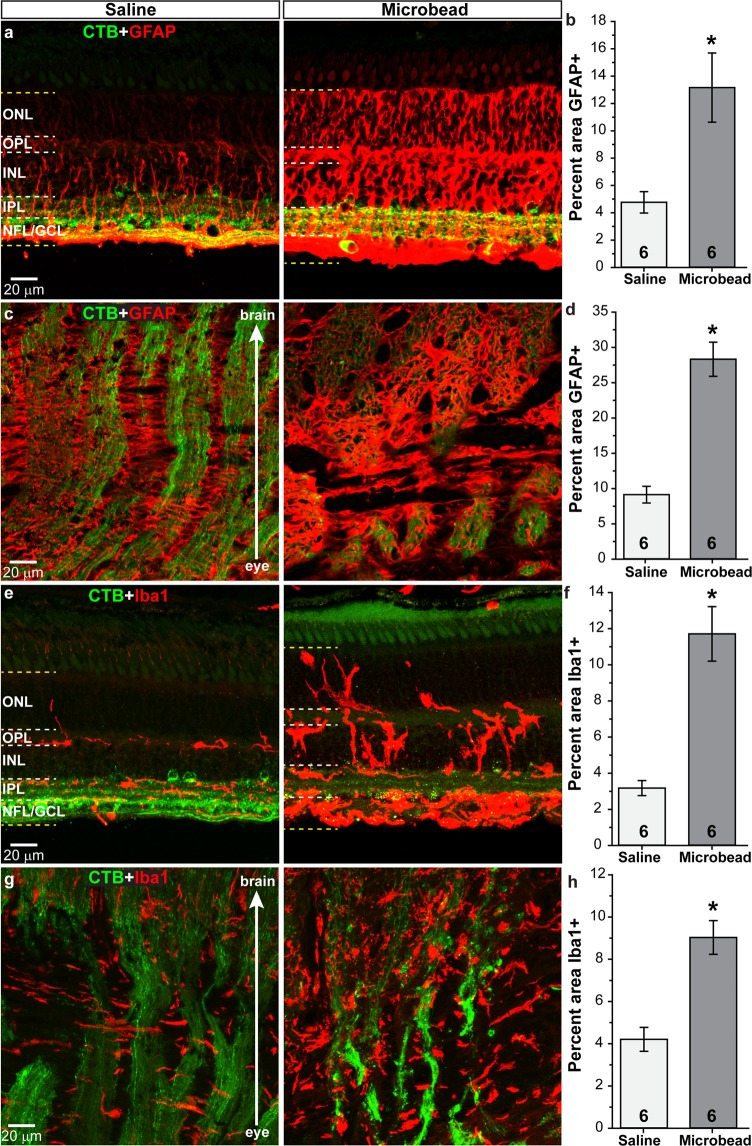
Elevated IOP increases glial markers in retina and optic nerve. Confocal images from saline- and microbead-injected SM eyes showing immunolabeling (red) for GFAP or Iba1 in the retina (**a**,**e**) and in the optic nerve (**c**,**g**). CTB (green) in RGC somas and axons is shown. NFL: nerve fiber layer; GCL: ganglion cell layer; IPL: inner plexiform layer; INL: inner nuclear layer; OPL: outer plexiform layer; ONL: outer nuclear layer. Label was quantified in region bounded by yellow dotted lines in retinal images. Bar graphs show percent area of image that was immunolabeled for GFAP in retina and optic nerve (**b**,**d**) and Iba1 in the retina and optic nerve (**f**,**h**). *p = 0.01 (**b**), p = 0.00003 (**d)**, p = 0.0003 (**f**), p = 0.0006 (**h**). n indicated in bars. Data are expressed as mean ± SEM. Statistical comparisons were made using two-sided t-tests.

Finally, we examined the expression of various proteins implicated in the pathogenesis of glaucoma in vertical retinal sections, with a focus on the mid-peripheral region of the retina which is similar to our analysis in rodents. We did not notice any differences in protein expression between the foveal region and other retinal regions. Glial fibrillary acidic protein (GFAP) is an intermediate filament expressed by astrocytes and by Muller cells in the retina. In retinas from saline-injected eyes, we observed GFAP (red) in the nerve fiber layer (NFL) and ganglion cell layer (GCL), around blood vessels, and in Muller cells and their endfeet (Fig. 4a). Following microbead injection, GFAP levels appeared to increase throughout the retina, from the NFL to the outer nuclear layer (ONL). Quantification of GFAP in the retina (Fig. 4b) shows microbead injection increased levels 176% compared to saline (p = 0.01, n = 6 for saline and microbead). Within the optic nerve head (ONH) of saline-injected eyes, we observed GFAP expression in astrocyte columns and in the lamina cribrosa; RGC axons transporting CTB (green) are seen as well (Fig. 4c). Labeling for GFAP appeared increased within the ONH following elevated IOP due to microbead injection, and quantification showed a 210% increase in GFAP levels in microbead-injected eyes compared to saline (Fig. 4d; p = 0.00003, n = 6 for saline and microbead).

Ionized Ca^2+^-binding adapter molecule 1 (Iba1) is a marker used to identify microglia^14,41,42^. We observed Iba1-positive microglia (red) in retinas from saline-injected eyes within the inner retina layers; cells appeared uniform in size with extended processes (Fig. 4e). Elevated IOP due to microbead injection appeared to increase Iba1 levels and microglia appeared larger with thickened processes. Microglia in microbead-injected retinas also appeared to span the INL and ONL and accumulate within the GCL. Similar to GFAP, Iba1 levels within the retina increased 269% following microbead injection compared to saline injection (Fig. 4f; p = 0.0003, n = 6 for saline and microbead). Microglia within the ONH of saline-injected eyes (Fig. 4g) were elongated and appeared to orient perpendicular to RGC axons (green) within the lamina cribrosa region before aligning parallel to RGC axon bundles in the optic nerve proper. Microbead injection appeared to increase levels of Iba1 within the ONH, and microglia appeared less elongated with more of an amoeboid morphology. Quantification of Iba1 within the ONH (Fig. 4h) shows microbead injection increased levels 114% compared to saline injection (p = 0.0006, n = 6 for saline and microbead). We did not observe a significant difference in GFAP or Iba1 levels in eyes from bilateral saline-injected SMs and the saline-injected eyes from saline/microbead-injected SMs (p > 0.129). This was also true for other markers we examined (p > 0.279).

**Figure 5:**
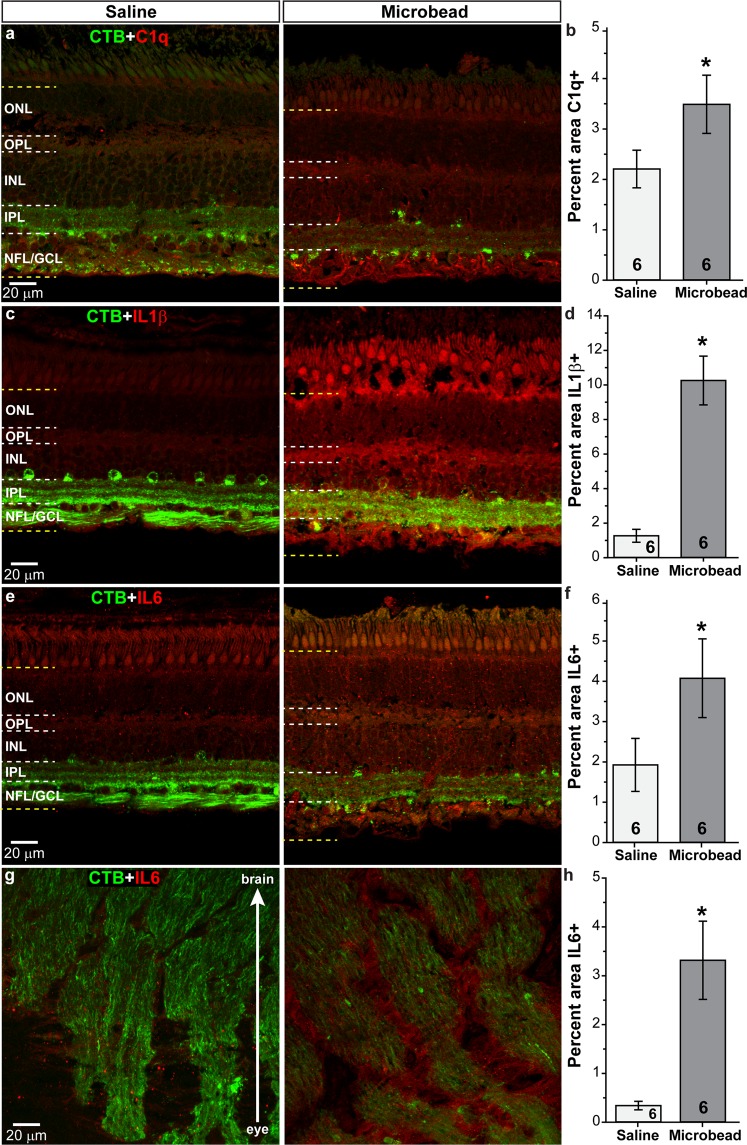
Elevated IOP increases neuroinflammatory markers. Confocal images from saline- and microbead-injected SM eyes showing immunolabeling (red) for complement 1q (C1q, **a**) or interleukin 1β (IL1β, **c**) in the retina, and interleukin 6 (IL6) in the retina (**e**) and optic nerve (**g**). CTB (green) in RGC somas and axons is shown. NFL: nerve fiber layer; GCL: ganglion cell layer; IPL: inner plexiform layer; INL: inner nuclear layer; OPL: outer plexiform layer; ONL: outer nuclear layer. Label was quantified in region bounded by yellow dotted lines in retinal images. Bar graphs show percent area of image that was immunolabeled for C1q (**b**), IL1β (**d**) in retina, and IL6 in the retina and optic nerve (**f**,**h**). *p = 0.046 (**b**), p = 0.0001 (**d**), p = 0.049 (**f**), p = 0.004 (**h**). n indicated in bars. Data are expressed as mean ± SEM. Statistical comparisons were made using two-sided t-tests.

Complement component 1q (C1q), an early component of the classical complement pathway, is upregulated in glaucoma, and may be involved in synapse loss within the retina^43,44^. Following microbead injection, levels of C1q (red) within the retina appeared elevated compared to saline injection (Fig. 5a); quantification confirmed a 58% increase in C1q levels in microbead-injected retinas compared to saline-injected retinas (Fig. 5b; p = 0.046, n = 6 for saline and microbead). We observed low levels of the neuroinflammatory mediators interleukin 1β (IL1β; Fig. 5c) and interleukin 6 (IL6; Fig. 5e) in the retina following saline injection^14,45–49^. Elevated IOP due to microbead injection appeared to increase IL1β and IL6 throughout the retinal layers, with intense labeling of the GCL layer (Fig. 5c,e). Quantification showed that retinal levels of IL1β were increased 708% (Fig. 5d), while IL6 increased 112% (Fig. 5f) compared to saline (p = 0.0001 for IL1β; p = 0.049 for IL6, n = 6 for saline and microbead). Microbead injection also upregulated IL6 within the ONH (Fig. 5g,h) showing an increase of 872% compared to saline injection (p = 0.004, n = 6 for saline and microbead).

**Figure 6:**
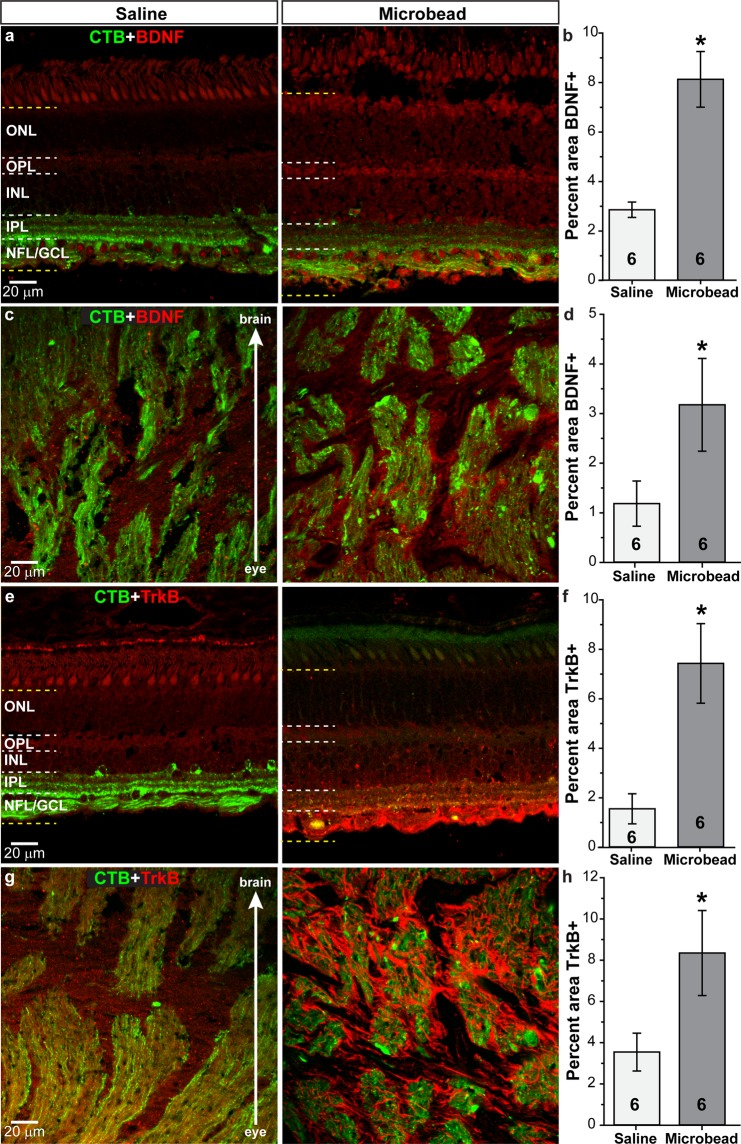
Elevated IOP increases BDNF and TrkB in the retina and optic nerve. Confocal images from saline- and microbead-injected SM eyes showing immunolabeling (red) for BDNF or TrkB in the retina (**a**,**e**) and in the optic nerve (**c**,**g**). CTB (green) in RGC somas and axons is shown. NFL: nerve fiber layer; GCL: ganglion cell layer; IPL: inner plexiform layer; INL: inner nuclear layer; OPL: outer plexiform layer; ONL: outer nuclear layer. Label was quantified in region bounded by yellow dotted lines in retinal images. Bar graphs show percent area of image that was immunolabeled for BDNF in retina and optic nerve (**b**,**d**) and TrkB in the retina and optic nerve (**f**,**h**). *p = 0.001 (**b**), p = 0.042 (**d**), p = 0.007 (**f**), p = 0.03 (**h**). n indicated in bars. Data are expressed as mean ± SEM. Statistical comparisons were made using two-sided t-tests.

We examined brain-derived neurotrophic factor (BDNF) and its receptor TrkB in SM eyes following IOP elevation as deprivation of neurotrophic factors, especially BDNF, may be involved in RGC degeneration in glaucoma^50–53^. In retinas from saline-injected SMs, BDNF (red) appeared to localize to RGCs in the GCL, axons or glia in nerve fiber layer (NFL) with diffuse staining throughout the INL and OPL (Fig. 6a). Within the ONH, BDNF localized to both RGC axons (green) and connective tissue elements (Fig. 6c). Elevated IOP appeared to increase BDNF levels in the retina, especially in the GCL and NFL, as well as in the ONH (Fig. 6a,c). Increases in BDNF were confirmed by quantification in the retina (Fig. 6b) and ONH (Fig. 6d), with levels 168 to 184% higher in microbead-injected eyes compared to saline-injected eyes (p = 0.001 for retina; p = 0.042 for ONH n = 6 for saline and microbead). TrkB (red) appeared as diffuse labeling throughout the retina following saline injection (Fig. 6e); in microbead-injected retinas, TrkB appeared increased in the NFL/GCL in processes that resemble astrocytes or Muller glia endfeet. We observed the diffuse pattern of TrkB label in the ONH of saline-injected eyes (Fig. 6g), and microbead injection appeared to increase TrkB expression in cells that resemble astrocytes or microglia. Quantification of TrkB in retina (Fig. 6f) shows a 378% increase following microbead injection (p = 0.007, n = 6 for saline and microbead), while a 135% increase was shown in the ONH (Fig. 6h; p = 0.03, n = 6 for saline and microbead).

**Figure 7:**
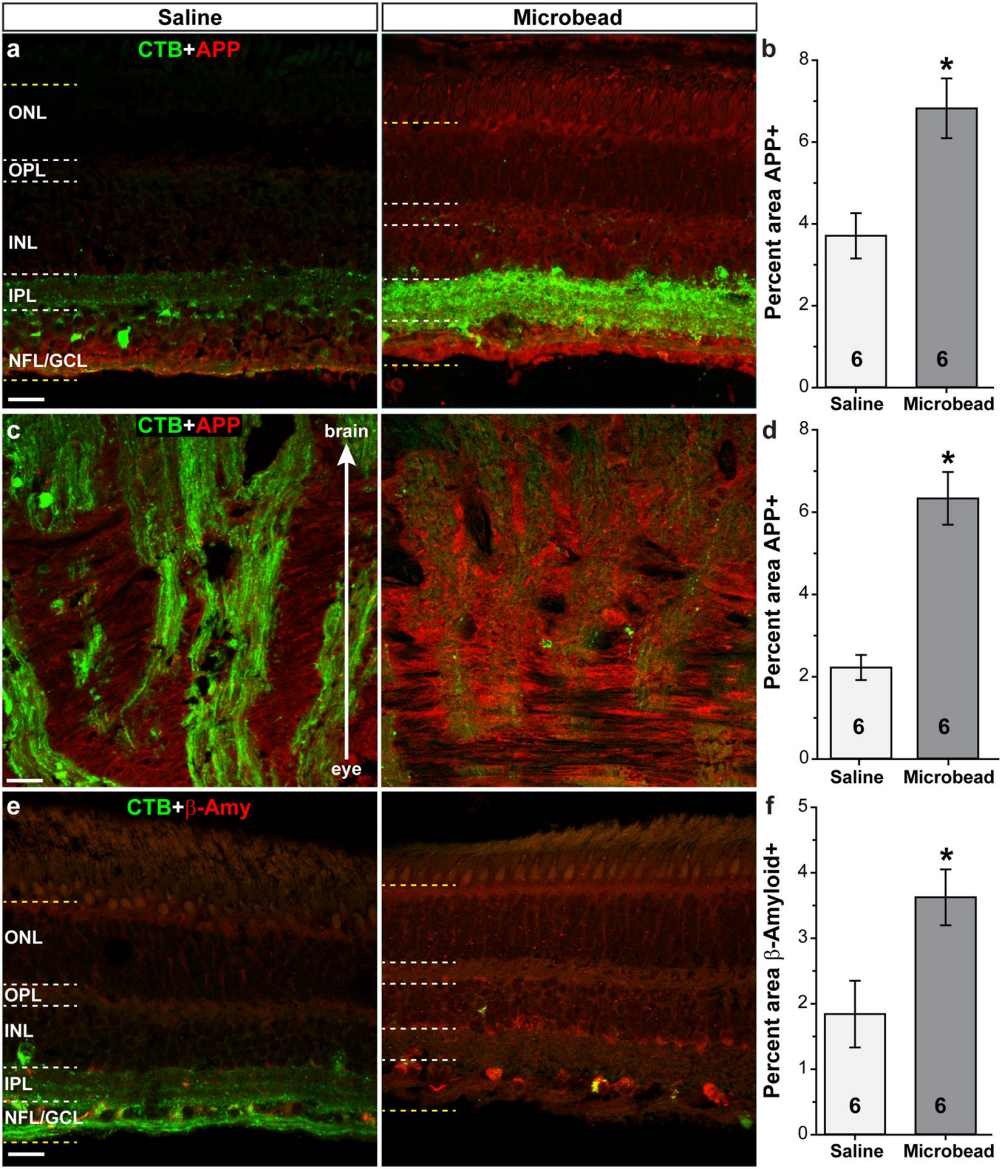
Elevated IOP increases proteins associated with neurodegeneration. Confocal images from saline- and microbead-injected SM eyes showing immunolabeling (red) for amyloid precursor protein (APP) in the retina (**a**) and optic nerve (**c**), and β-amyloid (β-amy) in the retina (**e**). CTB (green) in RGC somas and axons is shown. NFL: nerve fiber layer; GCL: ganglion cell layer; IPL: inner plexiform layer; INL: inner nuclear layer; OPL: outer plexiform layer; ONL: outer nuclear layer. Label was quantified in region bounded by yellow dotted lines in retinal images. Bar graphs show percent area of image that was immunolabeled for APP in the retina and optic nerve (**b**,**d**), and β-amyloid in retina (**f**). *p = 0.007 (**b**), p = 0.0002 (**d**), p = 0.02 (**f**). n indicated in bars. Data are expressed as mean ± SEM. Statistical comparisons were made using two-sided t-tests.

Amyloid precursor protein (APP) and its cleaved products (e.g. β-amyloid) have been implicated in glaucoma pathogenesis^54–56^. We observed APP (red) in retinas from saline-injected eyes within the inner retina layers, with little to no expression in outer retinal layers (Fig. 7a). Elevated IOP due to microbead injection appeared to increase APP levels throughout the retina; quantification shows APP levels within the retina increased 84% following microbead injection compared to saline injection (Fig. 7b; p = 0.007, n = 6 for saline and microbead). Within the optic nerve head (ONH) of saline-injected eyes, we observed APP in both RGC axons (green) and within glial columns and the lamina cribrosa (Fig. 7c). Labeling for APP appeared increased within the ONH following elevated IOP due to microbead injection, and quantification showed a 185% increase compared to saline (Fig. 7d; p = 0.0002, n = 6 for saline and microbead). In retinas from saline-injected eyes, we observed β-amyloid (β-amy; red) throughout the retinal layers (Fig. 7e). Following microbead injection, β-amyloid levels appeared to increase throughout the retina, especially in the GCL and INL. Quantification of β-amyloid in the retina (Fig. 7f) shows microbead injection increased levels 97% compared to saline (p = 0.02, n = 6 for saline and microbead). Also examined in retina were CD44 (Muller cell marker), MAP2 (dendrite marker), and phosphorylated Tau, and in ONH phosphorylated Tau and β-amyloid. Microbead injection had no effect on levels of these proteins compared to saline (p > 0.11 in retina, p > 0.2 in ONH, n = 6 for saline and microbead; Supplementary Table 1).

## Discussion

Here we present data from an inducible model of glaucoma for a non-human primate using intracameral injection of microbeads to elevate IOP. We trained SMs to accept awake IOP measurements in a restraint tube and elevated IOP by ~42% (+8.6 mm Hg) over the entire 36-week experiment using repeated microbead injection. We began to see a significant increase in IOP in microbead-injected eyes at 8 weeks post-initial injection, following 3 injections. In rodents, one injection of microbeads is sufficient to clog the majority of the trabecular meshwork, resulting in IOP elevation within 24 hours. We did not see significant IOP elevation in our SM model until 8 weeks after the first microbead injection, and after 3 injections. This delay in IOP elevation is most likely due to the size of the SM eye relative to the size of the microbeads. A similar delay in IOP elevation was noted by Weber *et al*. when injecting microbeads to elevate IOP in rhesus monkeys^4^. In this study, 50–100 μl of 10 μm microspheres were injected into the anterior chamber twice a week, with 8–10 injections needed to induce IOP elevations that were sustained 1–2 days. They observed that 2 to 3 injections resulted in small clusters of microspheres that did not fully obstruct the trabecular meshwork. After 20 injections they observed microspheres distributed across the inner surface of the trabecular meshwork, and subsequent IOP elevation to 24–37 mm Hg that were stable for up to 40 weeks^4^. We chose to inject microbeads every 3 weeks over a 15-week period for a total of six injections to allow time between injections for IOP to increase and to reduce stress and potential anesthesia-related effects for the animals. The time between injections could be reduced to elevate IOP faster as long as anesthesia-related effects, such as weight loss, were closely monitored in SMs. We observed SMs for three days post-microbead injection and at least 3 times a week over the course of the study to ensure against buphthalmia, conjunctival reddening, corneal ulcers, and/or signs of discomfort such as itching or rubbing of the eyes, eye closure, or excessive grooming of the face. We observed none of these signs, and SMs were active and engaged with cage mates and with veterinary or research staff. While visual field loss would be an expected outcome in a model of glaucoma, we did not observe impairment in moving about the cage, eating, drinking, interacting with cage mates, or any signs of discomfort or pain in SMs, indicating that vision loss was not complete or was at least subclinical. Future experiments utilizing this model could “fine-tune” the IOP elevation by reducing the number of injections and/or reducing the volume of microbeads, if so desired.

Other models used to elevate IOP in non-human primates include laser photocoagulation of the trabecular meshwork, intracameral injection of autologous ghost red blood cells, and topical dexamethasone treatment (steroid-induced glaucoma)^35,57–59^. Injection of red blood cells into the anterior chamber is similar to microbead injection, and induced IOP elevations range from 24–73 mm Hg and are sustained for up to 42 days^35^. While some corneal adverse effects were noted, no major inflammation was observed in the posterior eye and limited macrophage activity was seen in the anterior chamber^35^. Topical dexamethasone treatment is perhaps the simplest model in terms of equipment and/or training, yet its success rate is lower than other models as not every animal will respond to the steroid with elevated IOP. About 45% of cynomolgus monkeys treated with topical dexamethasone three times daily, every 3–4 days for 28 days responded with an average 11 mm Hg increase in IOP that returned to baseline levels within 2 weeks of stopping treatment^59^. Laser photocoagulation of the trabecular meshwork is the most popular method for elevating IOP in non-human primates and involves treating the trabecular meshwork with an argon laser^57,58^. Typically, more than one treatment is needed to induce IOP elevation, but stable elevations between 20–50 plus mm Hg can be achieved in as few as 4 weeks and last up to 260 days^58,60^. Ocular pressure can drop after treatment and laser-induced inflammation may occur^60^. Microbead injection in non-human primates requires limited equipment and minimal training, while producing IOP elevations comparable if slightly lower than laser photocoagulation. We obtained sustained IOP elevation for 28 weeks and, with reinjection as IOPs began to fall, could possible achieve elevations out to 36 months^4^. We observed no ocular inflammation following microbead injection in our SMs and observed no adverse effects to the eye or animal in general (see above). Together, these features suggest microbead intracameral injection to elevate IOP in non-human primates is an accessible model to investigate glaucomatous degenerative mechanisms and therapeutic interventions.

Microbead injection in SMs resulted in a mean IOP of 29.2 ± 1.2 mm Hg over 36 weeks, with an average IOP elevation of 52% from week 8 to the study end at 36 weeks (28 weeks sustained IOP elevation). Typically, mean IOP elevation is 25–35% over 4–6 weeks following microbead injections in rodents^17,32,34,61^. Given the larger elevation in SMs over a longer period, it was not surprising to see a greater loss of anterograde transport (54% in SMs vs. 36–45% in rodents), although RGC axon loss was similar to that observed in rodents (22.1% vs. 17–42%)^31,32,49,62^. In addition to loss of anterograde transport and degeneration of RGC axons in the optic projection, 28 weeks of elevated IOP in SM eyes induced upregulation of proteins implicated in glaucomatous neurodegeneration in the retina and ONH. Activation of astrocytes, Muller cells, and microglia in human glaucomatous retina and ONH samples and in experimental models of glaucoma is well recognized^42^. We showed increased levels of GFAP and Iba1 in the retina and ONH of SM eyes following elevated IOP due to microbead injection suggesting a similar activation of glia in our model. Along with glial activation, we observed an upregulation of C1q, a member of the complement pathway believed to mediate synapse loss in early stages of neurodegeneration, within the retinas from microbead-injected eyes^44^. Also increased in SM retina and ONH following microbead injection were BDNF and TrkB. While TrkB is upregulated in some experimental models of glaucoma, it is downregulated in human samples and other models^51–53^. BDNF expression during glaucoma progression is also variable^53^. Interestingly, IL1β is upregulated after CNS injury and during chronic neurodegeneration, and has been shown to increase BDNF, TrkB, and APP expression^63,64^. We observed increased expression of IL1β, IL6, APP and β-amyloid in SM eyes following IOP elevation. Each of these proteins are implicated in glaucoma pathogenesis, suggesting our model in SMs induces similar injury responses to other models^47–49,65,66^.

The results we present here are similar those shown in inducible glaucoma models using microbead injection in rodents^17,32,34,49,67^. This is an important point, as the ocular anatomy of rodents is different in key areas compared to primates. For example, rodents lack a macula and a fovea, a cone-rich region of maximal visual acuity present in primate retinas^68,69^. Rodents also lack a lamina cribrosa, the connective tissue structure within the ONH where initial damage to RGC axons in glaucoma is thought to occur. However, rodents do have an extensive glial lamina composed mainly of astrocytes, and data suggest the ONH region is the site of glaucomatous injury in rodents as well as primates^26,70–73^. Finally, as mentioned briefly above, the majority (>95%) of RGC axons in rodents cross to contralateral targets in the SC and other visual processing structures, while in primates an equal proportion of axons travel contralateral and ipsilateral to visual targets in the LGN and SC^37^. We believe the similarity in outcome measures presented here with results observed in rodent models and in human patients with glaucoma suggest microbead occlusion in SMs is an attractive model to investigate degenerative mechanisms and therapeutic interventions in glaucoma.

## Methods

### Animals

The Vanderbilt University Institutional Animal Care and Use Committee approved all experimental procedures, which meet the standards and guidelines set forth in the Animal Welfare Act and the Guide for the Care and Use of Laboratory Animals, Eighth Edition. We obtained adult, male Bolivian squirrel monkeys (*Saimiri boliviensis*), from the University of Texas MD Anderson Cancer Center (Bishop, TX). Animals were between 2 and 4 years of age and had no history of diabetes or head/eye trauma. SMs were socially housed, received environmental enrichment daily, and were maintained in a 12-hour light-dark cycle with standard primate biscuits and water available *ad libitum*.

### Intraocular pressure measurement in awake squirrel monkeys

We worked with the Vanderbilt University Apparatus Shop to design and build a custom restraint tube for awake intraocular pressure (IOP) measurements in SMs (Supplementary Methods, Supplementary Figs 1 and 2). We trained SMs to accept hand-catching, placement into the restraint tube, and awake tonometry measurements, including application of a topical anesthetic, using various positive-reinforcement training techniques, such as acclimation/desensitization and shaping goal behaviors. Animals were acclimated and desensitized to research personnel, the catch gloves, the restraint tube, and the tonometry instruments (TonoPen XL rebound tonometer; Medtronic Solan, Jacksonville, FL), as well as the sounds made by tonometry instruments. Training sessions occurred daily, Monday through Friday, and lasted 15–30 minutes per animal. Each training session ended with a food or drink reward approved by the Vanderbilt Department of Animal Care. Once trained, we performed IOP measurements (10 to 15 readings per eye per session) in awake SMs following administration of 0.5% proparacaine hydrochloride ophthalmic solution (Patterson Veterinary Supply, Inc.) as a local anesthetic. We measured awake IOPs weekly over the course of the study.

### Microbead intracameral injection

At least 16 hours prior to microbead injection, staff evaluated SMs for health, alertness and general well-being. At this time, we removed primate biscuits from cages and supplemented with a limited amount of produce for pre-anesthetic fasting. We anesthetized SMs using 6 mg/kg ketamine (100 mg/ml; Patterson) plus 0.025 mg/kg dexmedetomidine (0.5 mg/ml; Patterson) via intramuscular injection and maintained anesthesia with 2.5% isoflurane (Patterson Veterinary) administered via a pump (VetEquip, Livermore CA). We administered 0.01 mg/kg glycopyrrolate (0.2 mg/ml; Patterson) and 1 mg/kg Cerenia (maropitant citrate; 10 mg/ml; Zoetis, Parsippany, New Jersey) subcutaneously. We maintained SM body temperature by placing them on a heated surface (Gaymar T/P 650 warm water recirculator and multi-T pad TP-R22G; Kent Scientific Corp., Torrington, CT) and Deltaphase® Isothermal Pads (ASS7T 6” x 7.5”; Braintree Scientific, Inc.; Braintree MA). We monitored heart rate, respiratory rate, and specific oxygen percentage every 10–15 minutes using a SurgiVet® V1030 Hand Held Pulse Oximeter (Patterson Veterinary) attached to the animal’s footpad; we monitored body temperature using a commercially available digital thermometer. We washed the eyes in sterile saline solution (Fisher Scientific), dilated the iris using 1% tropicamide ophthalmic drops (Henry Schein® Animal Health), and provided pre-emptive anesthesia using 0.5% proparacaine ophthalmic drops. We injected 40 μl of 25–35 μm polystyrene microbeads (FP-30052–5; Spherotech, Inc., Lake Forest, IL) plus 1% Hydroxypropyl Methyl Cellulose (HPMC) in sterile phosphate buffered saline (PBS) into the anterior chamber using a 31-gauge needle attached to a syringe (Fisher Scientific); saline injected eyes received an equal volume of 1% Hydroxypropyl Methyl Cellulose (HPMC) in sterile PBS. We provided 0.25 mg/kg Antisedan (5 mg/ml; Patterson) via intramuscular injection to reverse the sedative and analgesic effects of dexmedetomidine. We monitored SMs during recovery and returned them to their home cage when able to maintain sitting and standing positions. Three monkeys received unilateral microbead injection (fellow eye received saline), four monkeys received bilateral microbead injection, and four monkeys received bilateral saline injection. Overall, SMs tolerated weekly awake IOP measurements and intracameral injections of saline or microbeads well. Three animals demonstrated moderate weight loss (0.12–0.16 kg, 14–18% body weight) during the experiment and, after consulting with Vanderbilt’s Department of Animal Care, SMs received daily dietary supplement in the form of Ensure® soaked biscuits. We observed no adverse effects from elevated IOP due to microbead injections. SMs were active, engaged in social interaction, and showed no signs of visual impairment.

### Anterograde transport analysis

We prepared, anesthetized and monitored SMs as described for microbead injection. We washed the eyes in sterile saline solution, and provided pre-emptive anesthesia using 0.5% proparacaine drops. We injected 40 μl of a 1% solution of cholera toxin beta subunit (CTB) conjugated to a fluorophore (ThermoFisher Scientific) into the vitreous cavity using 31-gauge needle attached to a syringe. Right eyes received CTB-Alexa 594 and left eyes received CTB-Alexa 488. Reversal of anesthesia and post-procedure monitoring are similar to that described above. Seven days after intravitreal injection of CTB, we sedated SMs with 20 mg/kg ketamine and 0.4 mg/kg xylazine (20 mg/ml; Patterson) via intramuscular injection. We euthanized SMs using 120 mg/kg sodium pentabarbitol (390 mg/ml, Euthasol; Patterson Veterinary) via intravenous injection and transcardially perfused with PBS followed by 4% paraformaldehyde in PBS. We cryoprotected brains in 30% sucrose in PBS and cut 52 µm coronal sections using a sliding microtome. We mounted alternating sections of LGN and SC and imaged sections using a Zeiss FV-1000 inverted confocal microscope through the Vanderbilt University Medical Center Cell Imaging Shared Resource. We used identical microscope settings to acquire images for quantification and comparison. We quantified the intensity of CTB-488 or CTB-594 signal in the SC and LGN using a custom macro in ImagePro (Media Cybernetics) that determines the percent area of the positive label^74^. We calculated the percent of intact transport for each section by totaling the area of tissue positive for CTB-488 and CTB-594, and then dividing by the total area of the section (CTB-488^+^ area + CTB-594^+^ area/total area). A minimum of 6 LGN sections/hemisphere/SM and 10 SC sections/hemisphere/SM were quantified. In one SM that received bilateral saline injection the SC region of the brain was torn during sectioning resulting in loss of SC tissue prior to analysis. This monkey was excluded from SC analysis resulting in a sample size of five SCs from saline-injected eyes. Transport from experimental (intracameral injection of saline or microbeads) was normalized to transport from naïve (no intracameral injections) SM eyes. CTB uptake by RGCs in the retina was verified using confocal microscopy of whole-mounted retinas.

### Optic nerve analysis

We isolated 3 mm sections of optic nerve (ON) proximal to the globe and post-fixed for 2 hours in 2% glutaraldehyde. We prepared ONs for epon embedding and semi-thin cross-sectioning as described previously^31,32^. We stained sections with 1% paraphenylenediamine (in a 1:1 mixture of methanol and 2-propanol) and 1% toluidine blue to identify myelin sheaths and glia, respectively. We imaged sections using 40x oil-immersion and differential interference contrast optics as a montage with a microscope equipped with a motorized X-Y-Z stage and a digital SLR camera (Nikon H600L and DS-Ri2). We counted axons using the AxonJ macro in Fiji^75,76^ and measured ON cross-sectional area using Fiji.

### Immunohistochemistry and image quantification

We dissected whole eyes and bisected the retina, preparing one half for whole-mount immunohistochemistry and the other half for cryoembedding and 10 μm vertical sectioning. We performed immunohistochemistry on cryosections and whole-mount retinas as previously described^12,32^. We used the following primary antibodies to immunolabel proteins of interest: amyloid precursor protein (1:50, MAB348, EDM Millipore); beta-amyloid (1:200, #2452, Cell Signaling Technologies); brain derived neurotrophic factor (1:100, ANT-010, Alomone Labs); complement component 1q (1:1000, A301, Quidel Corp.); glial fibrillary acidic protein (1:500, MAB360, EDM Millipore); interleukin 1beta (1:50, NB-600-633, Novus), interleukin 6 (1:400, ab6672, AbCam), ionized Ca2 + binding adaptor molecule 1 (1:400, ab 5076, AbCam), TrkB (1:300, 610101, BD Transduction Labs). Sections and whole-mount retinas were incubated with appropriate secondary antibodies (1:200; Jackson ImmunoResearch Laboratories, Inc.) and coverslipped with DAPI Fluoromount G (vertical sections) or Fluoromount G (whole-mounted retinas) (Southern Biotech, Birmingham, AL). We imaged cryosections and whole-mounted retinas using a Zeiss FV-1000 inverted confocal microscope. We took images in the mid-peripheral region of the retina for all vertical sections, and used identical microscope settings to acquire images for quantification and comparison. A naïve observer quantified immunolabel in sections using a custom macro in ImagePro (Media Cybernetics) that determines the percent area of the positive label^74^. For Figs 4–7, CTB in right eyes was pseudocolored green and protein of interest label was pseudocolored red for visual comparison.

### Statistical analysis

All data are expressed as mean ± standard error unless indicated otherwise. The number of samples used in each experiment is provided in the appropriate results section or figure legend. Statistical comparisons between two independent measurements were made using two-sided t-tests, following confirmation of normality for each using the Shapiro-Wilk normality test; samples for which normality failed were compared using the Mann-Whitney Rank Sum Test (SigmaPlot 11.1, Systat Software, Inc., Chicago, IL).

## Supplementary information

Supplementary Information

## Data Availability

All data generated or analyzed during this study are included in this published article.
