# Supplementary material for: Towards A Microbead Occlusion Model of Glaucoma for a Non-Human Primate
Source: Sci Rep. 2019 Aug 9;9:11572. doi: 10.1038/s41598-019-48054-y (PMC6689098; doi:10.1038/s41598-019-48054-y)
Supplement: Supplementary file 1 — Supplementary Information [file 41598_2019_48054_MOESM1_ESM.docx]

**Supplementary material for:**

**Towards A Microbead Occlusion Model of Glaucoma for a Non-Human Primate**

Wendi S. Lambert, Brian J. Carlson, Purnima Ghose, Victoria D. Vest, Vincent Yao, and David J. Calkins*

The Vanderbilt Eye Institute, Vanderbilt University Medical Center, Nashville, TN 37232-0654

**Supplementary Methods: Specifications for Restraint Tube**

We built a custom restraint tubes for adult SMs using the following specifications. The base was ½-inch (1.27 cm) polyvinyl chloride (PVC) sheeting 38 cm x 38 cm, with a ¼-inch (0.635 cm) thick circular base (15.87 cm, 15.24 cm OD/ID) glued to the center (Supplementary Figure 1, base). The body was ¼-inch (0.635 cm) thick PVC tubing, 38 cm tall, 13.97 cm OD, 13.3 cm ID (Supplementary Figure 1, body and top down view). The body has a cutout (9.84 cm x 15.87 cm) 17.78 cm from the top, cutouts for the neck plates 36.19 cm from the bottom, and holes (1.3 cm ID) at 12.7 cm, 15.24 cm and 17.78 cm from bottom for a perch (Supplementary Figure 1, body). The door (11.43 cm x 17.78 cm) is ¼-inch thick PVC tubing that slides into a PVC track glued onto the outside of the body (Supplementary Figure 2). Two neck plates 10.16 cm x 12 cm (short side) and 19 cm (long side) were cut from ¼-inch thick PVC sheets and placed inside the neck plate cutouts to allow plates to slide closed with the smallest diameter of 3.8 cm (Supplementary Figure 1 and 2). The perch is composed of three brushed aluminum rods 0.9 cm in diameter, 18.41 cm long held together by a bolt (Supplementary Figure 2).

**Supplementary Figure 1**

**
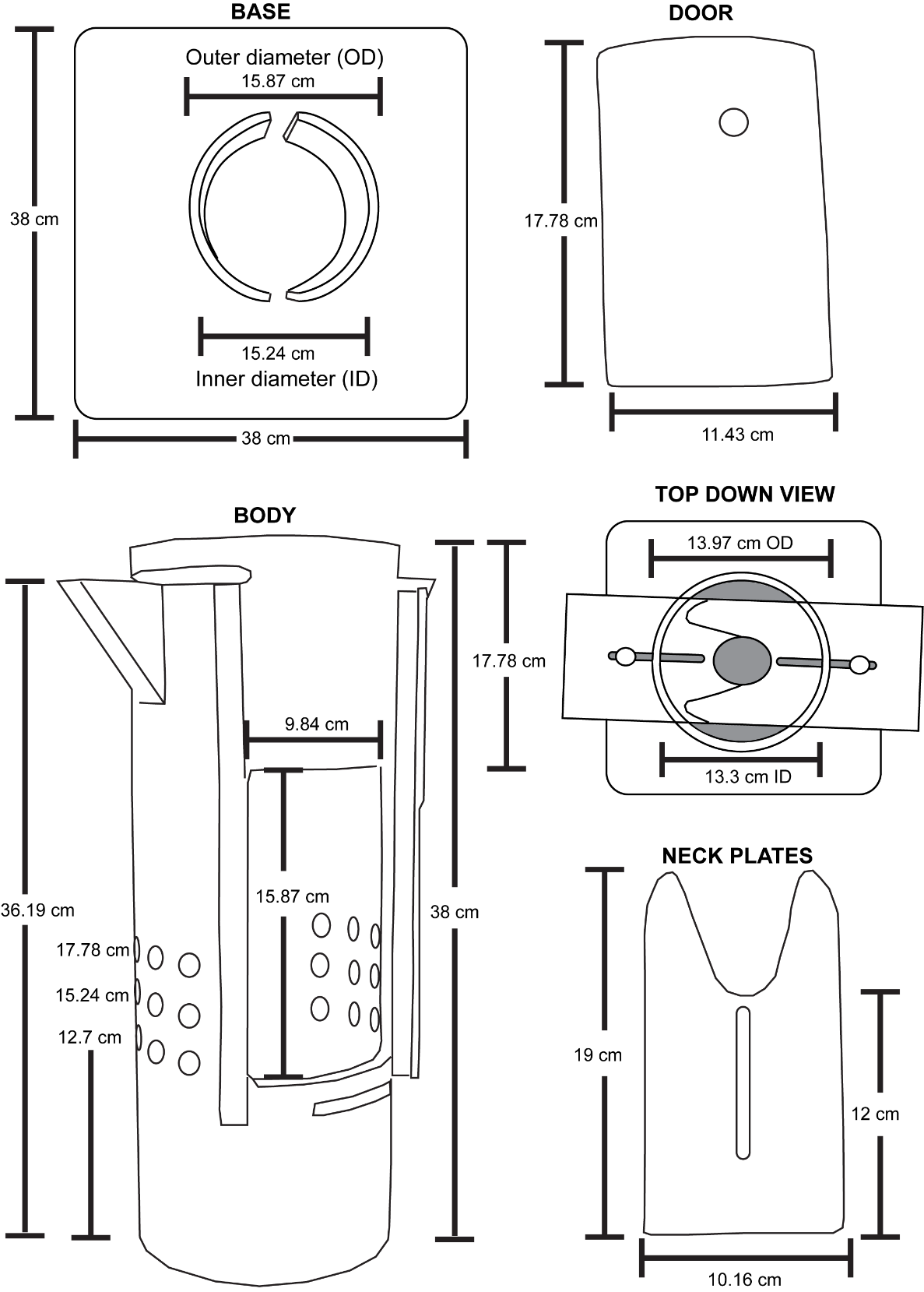
**

**Supplementary Figure 1.** Schematics for squirrel monkey restraint tube. Dimensions and measurements accounted for average adult squirrel monkey heights and weights, and for natural standing positions.

**Supplementary Figure 2**

**
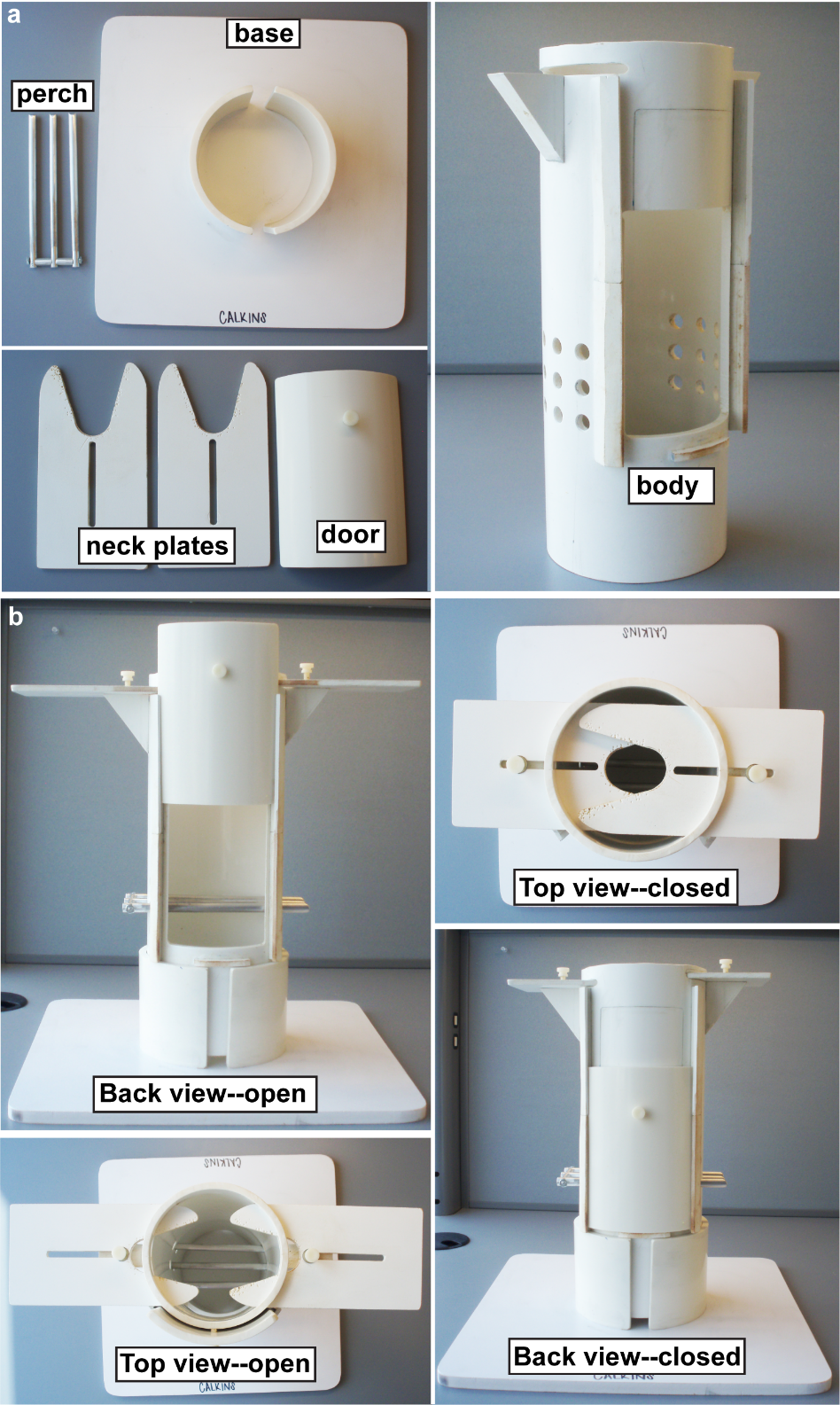
**

**Supplementary Figure 2.** Restraint tube for awake intraocular pressure measurements. (**a**) Components of a custom restraint tube made from polyvinyl chloride (PVC) sheeting or tubing and brushed aluminum rods. (**b**) Assembled restraint tube that allows for measurement of awake intraocular pressure in squirrel monkeys. Monkeys have full rotational ability in closed tube.

**Supplementary Table 1. Non-significant changes in protein expression**

Retinal Expression: Saline Microbead P value

CD44 4.69 ± 0.91 2.75 ± 0.62 0.11

MAP2 28.61 ± 4.44 30.06 ± 4.25 0.82

pTau 8.13 ± 1.01 9.69 ± 0.93 0.28

ONH Expression: Saline Microbead P value

β-amy 0.85 ± 0.20 1.39 ± 0.36 0.22

pTau 2.41 ± 0.46 3.95 ± 0.74 0.10

n = 6 for each group.
